# XFELs for structure and dynamics in biology

**DOI:** 10.1107/S2052252517005760

**Published:** 2017-05-10

**Authors:** J. C. H. Spence

**Affiliations:** a Department of Physics, Arizona State University, Tempe, AZ 85287-1504, USA

**Keywords:** X-ray lasers, XFELs, biology, structure, dynamics

## Abstract

The development and application of the free-electron X-ray laser (XFEL) to structure and dynamics in biology since its inception in 2009 are reviewed. The research opportunities which result from the ability to outrun most radiation-damage effects are outlined, and some grand challenges are suggested. By avoiding the need to cool samples to minimize damage, the XFEL has permitted atomic resolution imaging of molecular processes on the 100 fs timescale under near-physiological conditions and in the correct thermal bath in which molecular machines operate. Radiation damage, comparisons of XFEL and synchrotron work, single-particle diffraction, fast solution scattering, pump–probe studies on photosensitive proteins, mix-and-inject experiments, caged molecules, pH jump and other reaction-initiation methods, and the study of molecular machines are all discussed. Sample-delivery methods and data-analysis algorithms for the various modes, from serial femtosecond crystallo­graphy to fast solution scattering, fluctuation X-ray scattering, mixing jet experiments and single-particle diffraction, are also reviewed.

## Abstract 2

The use of X-ray lasers to obtain structures and molecular movies in biology is reviewed. Methods include single-particle imaging, serial crystallography and fast solution scattering at room temperature.

## 1. Background   

The first hard X-ray free-electron laser (XFEL), the Linac Coherent Light Source (LCLS), began operation at the US Department of Energy SLAC laboratory near Stanford in 2009 (Pellegrini, 2012 ▸). A second started at the Japanese SACLA laboratory in 2012 (Ishikawa *et al.*, 2012 ▸). Preliminary operation of XFELs in Korea (PAL) and Switzerland (SwissFEL) commenced in late 2016, while beamtime proposals for the European XFEL (EuXFEL) at the DESY laboratory in Hamburg, Germany were accepted early in 2017. This has created many new opportunities for crystallography and imaging at atomic resolution on timescales from femtoseconds to seconds. More importantly for structural biology, it has allowed time-resolved diffraction at room temperature, while avoiding most effects of radiation damage, in addition to allowing the study of submicrometre crystals that are too small for conventional crystallography. The ‘diffract-then-destroy’ method used outruns damage by using, for example, X-ray pulses of 40 fs in duration to produce an X-ray diffraction pattern before the onset of secondary damage from the growing cascade of photoelectrons, which subsequently destroys the sample. This has allowed ‘molecular movies’ (defined in more detail below) to be made at atomic resolution using microcrystals at room temperature, without the need for cooling to avoid damage. This may be performed in the correct thermal bath in which these molecular machines operate, under near-physiological conditions. [While the volatile buffer present in crystals may differ from the working environment of a protein, many enzymes remain active in the crystalline form (Hajdu *et al.*, 1988 ▸).] Since each sample is destroyed by a pulse, a continuously refreshed supply of hydrated microcrystals is therefore needed, running in single file across the pulsed beam. Their diffraction patterns must then be merged in this serial femtosecond crystallography (SFX) technique.

A typical pulse at the LCLS may contain about 10^11^ photons (at 8 kV). This will scatter more than a million photons from a single large virus, so that diffraction from single particles (SP) is also possible. X-ray beam diameters can be as small as 0.1 µm, with a fractional energy spread in the beam of 10^−3^ and a repetition rate for X-ray pulses of 120 Hz, limited by the read-out rate of the detector. At present (as discussed below), for scattering from a single virus, the resolution after three-dimensional reconstruction (requiring a large number of shots) is about 10 nm. Scattering occasionally extends to much higher resolution in individual shots, which currently do not provide sufficient data for three-dimensional reconstruction. Bragg reflections, however, are routinely detected from protein microcrystals at 0.2 nm resolution. Whereas a cubic micrometre of cryocooled protein crystal scatters about a million photons into Bragg beams by the time it has absorbed the critical damage dose of 30 MGy (Owen *et al.*, 2006 ▸), a much larger dose can be applied in a pulse which outruns damage (Barty *et al.*, 2012 ▸).

Chemical reactions such as enzyme catalysis involve time-dependent conformational changes in proteins, in addition to the static molecular shapes, constrained by crystal formation, that crystallography provides. The field of time-resolved crystallography (Moffat, 2014 ▸; Schlichting *et al.*, 1990 ▸) allows these atomic motions to be studied if they are compatible with the crystal lattice. The reacting species can be diffused into a crystal (which may take much longer than in solution) and the results of this mixing can be studied, with the advantage of a much higher spatial resolution than that obtained using solution scattering. We will see that the ability of the XFEL to use micrometre-sized crystals allows a considerable improvement in time resolution owing to the reduced diffusion times for substrates into microcrystals. Molecular dynamics can also be imaged using cryo-EM, using samples rapidly quenched from an equilibrium ensemble to provide images which may be sorted by conformational similarity, and thus displayed as a movie. Light-sensitive proteins may also be subjected to an optical flash during quenching, with millisecond quench times.

For crystals, the ‘Bragg boost’ is a powerful effect, since the intensity at the Bragg peak (not the angle-integrated intensity) which brings the peak above the noise level is proportional to the square of the number of molecules in the crystal, so that even a nanocrystal consisting of 10 × 10 × 10 molecules will provide a million times more peak intensity than one molecule. (Background, pixel size and other scattering artifacts may complicate this simple estimate.)

The field of XFEL applications to structural biology has been reviewed by Bostedt *et al.* (2016 ▸), Spence (2017*b* ▸) and Schlichting (2015 ▸), in a special issue of *Philosophical Transactions* (Spence & Chapman, 2014 ▸), in a special issue of *Structural Dynamics* (Ourmazd, 2015 ▸) and by Spence *et al.* (2012 ▸). A simple explanation of the operation of the XFEL can be found in Ribic *et al.* (2012 ▸), and the history of the invention of the XFEL has been reviewed by Pellegrini (2012 ▸). A compact XFEL, smaller than a campus laboratory, is under construction at Arizona State University in collaboration with MIT (Graves *et al.*, 2012 ▸), and a compact attosecond XFEL is planned at the DESY laboratory in Hamburg, Germany (Kärtner, 2016 ▸).

## 2. Experimental methods for XFEL structural biology   

It is convenient to classify research in structural biology at XFELs according to the techniques used. These XFEL data-collection modes include fast solution scattering (FSS), the use of protein microcrystals for serial femtosecond crystallo­graphy (SFX) and single-particle (SP) imaging (with one particle per shot), among others. The term ‘nanocrystal’ has been used rather loosely in the literature: we suggest that crystals larger than one micrometre in size (largest dimension) be referred to as ‘microcrystals’. The majority of SFX studies published have used crystals of a few micrometres in size, but diffraction from crystals with less than 50 molecules on a side has occasionally been seen.

**Figure 1:**
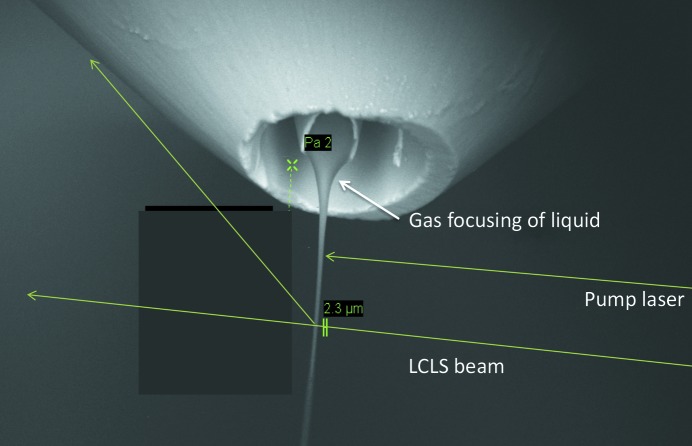
Environmental SEM image of a hand-ground gas dynamic virtual nozzle (GDVN) system. The liquid can be seen to narrow as the outer jacket of high-pressure gas speeds it up as it enters vacuum at about 10 m s^−1^, where it breaks up into droplets which freeze at about 10^6^°C s^−1^ (Weierstall *et al.*, 2012 ▸; image courtesy of D. DePonte). A Bragg beam is indicated, scattered from a microcrystal in the stream to the top left, and a pump laser is also shown for use with light-sensitive proteins.

The history and invention of the most popular mode at present (SFX) can be traced to early proposals for the delivery of samples across a beam by liquid jet (Spence & Doak, 2004 ▸) and to the first applications and development of this method at a synchrotron (Shapiro *et al.*, 2008 ▸) in preparation for its use in the first crystallography experiments at the LCLS (Chapman *et al.*, 2011 ▸) using a gas-dynamic virtual nozzle (GDVN) for sample delivery (Weierstall *et al.*, 2012 ▸). In this approach, femtosecond X-ray pulses diffract from successive hydrated microcrystals, running in single file across the focused XFEL beam in random orientations, as shown in Fig. 1 ▸. Each microcrystal is destroyed by the beam following diffraction. Diffraction patterns are read out at 120 Hz at the LCLS. The GDVN nozzles, which provide a fast coaxially flowing gas to focus the liquid, and so avoid clogging, can now be fabricated by two-photon laser printing with submicrometre resolution. This method of nozzle formation (Nelson *et al.*, 2016 ▸) opens up new possibilities for testing prototypes for all sample-delivery modes, including mixing jets and sheet jets for fast solution scattering (FSS).

**Figure 2:**
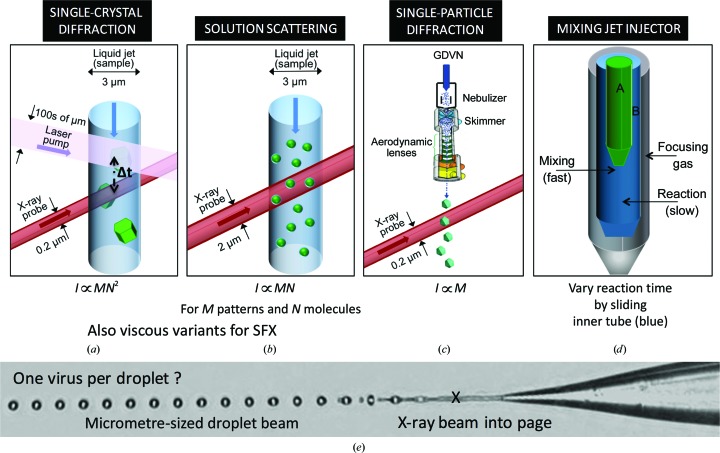
(*a*) Serial femtosecond X-ray diffraction (SFX) with one protein microcrystal per shot. (*b*) Fast solution scattering (FSS) with many similar molecules per shot. (*c*) The single-particle (SP) mode with one particle per shot. (*d*) A mixing jet for snapshot imaging of slow dynamics. (*e*) shows a simple Rayleigh jet, in which the water stream breaks up into perfectly spherical droplets a few micrometres in diameter (Weierstall *et al.*, 2008 ▸). Fixed-sample stages are also used (see text). All these may be combined with X-ray absorption or emission spectroscopy.

If we add to these methods the possibility of mixing solutions together for a chemical reaction prior to injection, we obtain the four common experimental methods summarized in Fig. 2 ▸. They are serial femtosecond crystallography (SFX), with one protein microcrystal per shot, fast solution scattering (FSS) (or ‘snapshot WAXS’), single-particle diffraction (SP) with one particle, such as a virus, per shot, and mix-and-inject studies for snapshot imaging of chemical reactions (using either solution scattering or microcrystals). Other delivery modes, such as viscous media ‘toothpaste’ jets, such as a lipid cubic phase (LCP) jet (Weierstall *et al.*, 2014 ▸), or one based on mineral oil (Sugahara *et al.*, 2015 ▸) have been described, including conveyor belts supplied with droplets (Fuller *et al.*, 2017 ▸) and ‘fixed-target’ methods, in which samples are scanned across the beam, usually in two dimensions, as described below. The various sample-delivery methods have been compared in Weierstall (2014 ▸). Because of the high speed (about 10 m s^−1^) of the GDVN liquid jet, most protein microcrystals run to waste between shots with a 120 Hz repetition rate. In order to conserve precious protein, viscous jets were developed (Weierstall *et al.*, 2014 ▸; Botha *et al.*, 2015 ▸; Conrad *et al.*, 2015 ▸) using a medium with the high viscosity of automobile grease, which emerges slowly from the nozzle. The viscous LCP medium also has the great advantage that it forms a growth medium for membrane-protein microcrystals, such as G-protein coupled receptors (GPCRs; Landau & Rosenbusch, 1996 ▸), so that crystals can be grown and delivered in the same system. Details of the construction of the LCP jet are given in Weierstall *et al.* (2014 ▸).

While most time-resolved SFX has been undertaken using the simple but wasteful GDVN arrangement (which accepts both membrane and soluble proteins), there has been some success using viscous jets to collect time-resolved data. The grease-matrix injector (Sugahara *et al.*, 2015 ▸) has been used to image the site of oxygen-bond formation in photosystem II (Suga *et al.*, 2017 ▸) by time-resolved SFX. The use of the LCP jet for time-resolved diffraction at LCLS was reported by Nogly (2016 ▸), and used for a multi-frame three-dimensional movie of bacteriorhodopsin by Nango *et al.* (2016 ▸). The use of this approach at higher repetition rates remains to be determined, since the shock wave generated during the destruction of one sample in the liquid tube must not interfere with the next microcrystal. This constrains the distance between crystals, the flow velocity and the intensity of the X-ray pulse, as shown in the dramatic images of exploding jets by Stan *et al.* (2016 ▸).

While the liquid jets are best suited to the high repetition rates expected in future XFELs, with their faster cameras, and can be used for the structure determination of soluble proteins which may not grow in LCP, a number of ‘fixed-target’ systems have been developed which are much less wasteful in their use of protein. These include particle-trap arrays on chips (Lyubimov *et al.*, 2015 ▸) and scanned arrays (Mueller-Werkmeister *et al.*, 2010 ▸; Oghbaey *et al.*, 2016 ▸) for both three-dimensional and two-dimensional crystals (Frank *et al.*, 2014 ▸). One recent system (Roedig *et al.*, 2016 ▸) uses a silicon membrane with small holes that are slightly larger than the protein microcrystals. The crystals are ‘wicked’ down by a filter paper on the underside of the silicon membrane into the holes from solution above, and become jammed in the holes. The first-order (lowest angle) Bragg diffracted beams from the single-crystal wafer are diffracted to a high angle (possibly beyond the edge of the detector), so that the background is very low, since inelastic X-ray scattering between Bragg reflections in silicon is very weak and owing to the absence of solvent background. The crystals are kept hydrated with flowing wet helium gas, and the data are collected in air or a helium environment at atmospheric pressure. This promising ‘road-runner’ arrangement is under further development for time-resolved diffraction. A second scanned fixed-sample arrangement, which uses spectroscopy to locate the microcrystalline samples, with a high hit rate, is described in Oghbaey *et al.* (2016 ▸). Fixed samples have also been used by Hirata *et al.* (2014 ▸) to collect data from large single crystals.

For single particles, most sample delivery has used the aerodynamic lens stack gas-focusing injector system (Hantke *et al.*, 2014 ▸), driven by a GDVN or electrospray, as extensively developed by the Hajdu group in Uppsala. Whereas hit rates for LCP injectors may be higher than 40% for microcrystals, it has proven very difficult to obtain a hit rate above about 1% for single particles in these systems. The hit rate is 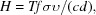
where *T* is a transmission coefficient for the particle jet (to account for the loss of particles in transit to the nozzle), *f* is the particle-injection frequency, σ is the sum of the X-ray beam diameter and the particle diameter, υ is the XFEL repetition rate, *c* is the particle speed and *d* is the particle-beam diameter, which is assumed to be larger than the X-ray beam diameter. We see that the hit rate can be increased most readily by increasing the injection frequency, increasing the repetition rate or reducing the particle speed.

The use of viruses lying on a hydrated graphene substrate may have advantages. A simple convergent nozzle has given a 2 µm focus of 200 nm particles (Kirian, Awel *et al.*, 2015 ▸), while the possibility of running viruses along a hollow tube of light (a Bessel beam) has also been explored (Eckerskorn *et al.*, 2013 ▸). New optical imaging methods now allow, for the first time, bioparticles to be directly observed during injection at XFELs, a most important advance to assist alignment (Awel *et al.*, 2016 ▸).

Finally, the double-focusing system, further described in §[Sec sec7]7, is also shown in Fig. 2 ▸ (Wang *et al.*, 2014 ▸). An important development has been the use of this arrangement for static structure determination (SFX) using ethanol as the second fluid, instead of the substrate used in the mix-and-inject studies described in §[Sec sec7]7 (Oberthuer *et al.*, 2017 ▸). A third outer coaxial fast gas jacket is also used to focus the two liquid streams. It is found that the diameter of the innermost stream carrying the microcrystals can be reduced to zero by adjusting the speed of the intermediate alcohol stream, so that this system acts as a smooth cutoff valve, reducing sample consumption as required. This system appears to be the most generally applicable to the widest range of conditions at present, since it supports both soluble and membrane proteins, conserves protein, can be used for fast time-resolved pump–probe (or mixing) experiments and will operate up to the megahertz repetition rates expected for future XFELs. The temperature at the nozzle should be controllable in the future.

Since all of these modes have time-resolved variants, the full taxonomy of data-collection modes might be labeled SFX, FSS, SP, TR-SFX, TR-FSS and TR-SP. The time-resolved modes may use a variety of means to initiate reactions, including optical pulses (for example in the study of light-sensitive proteins), chemical mixing or applied electric fields. Only TR-SP can provide a true molecular movie without any form of ensemble averaging or modeling.

## 3. Radiation damage and resolution   

Radiation damage places a fundamental limit on resolution in practically all imaging and diffraction methods in biology (with the exception of neutron diffraction). Single-particle (SP) cryo-electron microscopy (cryo-EM) deals with this by merging many real-space images of similar particles, each of which receives less than the very low critical ‘damage dose’, which is too small to allow a useful image to be formed from one molecule alone. This dose is a function of resolution, in which high-resolution detail (corresponding to higher angle scattering) is destroyed first during exposure: thus, the high-order Bragg reflections fade before the low orders during an extended synchrotron exposure, so that radiation damage has a similar effect initially to an increase in temperature. Detailed measurements and theory for the resolution-dependence of dose are given in Howells *et al.* (2009 ▸). By comparison with similar XFEL SP data-merging algorithms, the cryo-EM real-space images present no phase problem, and do not possess the additional Friedel symmetry which is present in diffraction patterns. Imaging thus solves the phase problem.

Breedlove & Trammel (1970 ▸) showed that single-atom imaging of molecules should never be possible using any form of scattered radiation (except perhaps neutrons and He atoms, for which bright sources did not exist), because the radiation dose needed to do so would destroy the molecule. This follows from the ratio of cross-sections for useful image-forming elastic scattering to damaging inelastic scattering over a range of beam energies and types of radiation. A fuller analysis (Henderson, 1995 ▸) used this ratio multiplied by the average amount of energy deposited in the sample by inelastic scattering as a figure of merit to compare damage and resolution for electrons and X-ray diffraction (XRD). This average deposited energy is about 20 eV for transmission electron microscopy (TEM) and is approximately equal to the full X-ray beam energy for XRD, where photoelectrons are created by an inelastic event in which the photon is annihilated. (In TEM, following an energy-loss event, the beam electron continues to the detector with lower energy to produce background in the diffraction pattern.) Since both the scattering cross-section ratio and the amount of energy deposited favor electrons, Henderson concluded that electron microscopy provides more information per unit damage than X-ray diffraction.

The Breedlove paper also contains the sentence ‘…this does not prevent X-ray molecular microscopy if the observations are made sufficiently rapidly…within 10^−13^ s’. This estimate of 100 fs for damage-free imaging has turned out to be remarkably prescient: recent XFEL crystallography using 50 fs pulses has shown 0.2 nm resolution scattering at huge doses from crystals and 0.59 nm scattering from individual virus particles at the LCLS, summed over many shots (Munke *et al.*, 2016 ▸). This idea that one could ‘out-run’ radiation damage was then further explored by Solem (1986 ▸) and, in detail, in response to the promise of the XFEL with its high-intensity femtosecond pulses, in molecular-dynamics simulations by Neutze *et al.* (2000 ▸). For a review, see Chapman *et al.* (2014 ▸). [Recall that X-rays are scattered by the atomic electron cloud alone, rather than the nuclei, whose positions are tracked in molecular-dynamics simulations. Electron beams are scattered by both electrons and nuclei. A comparison of fast X-ray and electron diffraction for the purposes of out-running radiation damage is given in Spence (2017*a* ▸).] Since the accumulation of damage processes in crystallography occur on timescales as long as a second (Hendrickson, 1976 ▸; *e.g.* bubble formation), the idea of out-running damage was not entirely new, but the conceptual breakthrough here was to realise that if laser amplification allowed an almost unlimited number of X-ray photons to be packed into a arbitrarily brief pulse, one could break the nexus between resolution, radiation damage and sample size (Howells *et al.*, 2009 ▸) and thus, in principle, achieve damage-free atomic resolution from arbitrarily small samples, such as a single virus, if a beam could be focused down to these dimensions. One could also study samples in conditions close to their native, room-temperature environment, avoiding the need to freeze samples to reduce damage. The first experimental evidence for this ‘diffract-then-destroy’ mechanism came at lower resolution using the VUV laser Flash at DESY in 2006 (Chapman *et al.*, 2006 ▸), suggesting the possibility of high-resolution, almost damage-free ‘movies’ (Spence, 2008 ▸). High-resolution (0.8 nm) results from protein nanocrystals and microcrystals using a 1.8 kV XFEL beam were first published in 2011 (Chapman *et al.*, 2011 ▸), together with the first single-particle XFEL results (Seibert *et al.*, 2011 ▸). Following initial elastic scattering, for samples larger than the inelastic mean free path of ejected photoelectrons, the photoelectrons thermalize, taking the sample temperature to perhaps 500 000 K and vaporization. For samples smaller than this size, the photoelectrons escape, leaving a charged sample which undergoes a Coulomb explosion.

**Figure 3:**
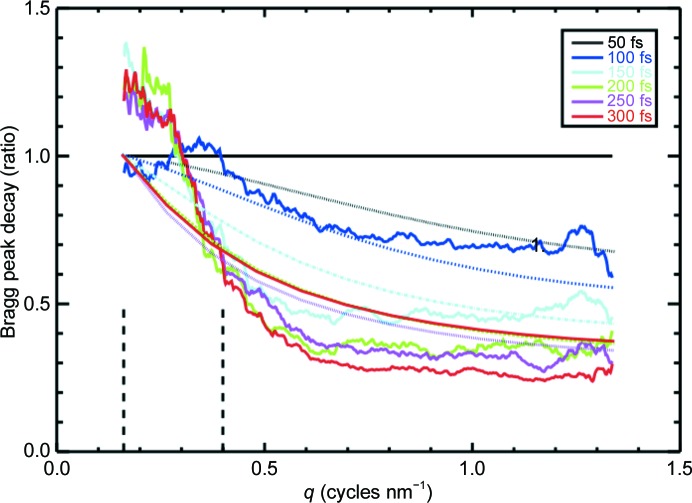
Bragg peak intensity in merged SFX from photosystem I (Chapman *et al.*, 2011 ▸) as a function of inverse resolution (in nm^−1^) for several different X-­ray pulse durations, normalized to the result at 50 fs (Barty *et al.*, 2012 ▸). Fine detail is destroyed first, and the effective pulse duration is set by the time taken to attenuate the high-order Bragg peaks.

Fig. 3 ▸ shows the fading of high-angle scattering (corresponding to the finest detail in the sample) with increasing XFEL pulse duration at 1.8 kV for Bragg diffraction from photosystem I (Barty *et al.*, 2012 ▸). For the longest pulses, late-arriving X-rays are diffracting from a crystal that is already damaged. The incident pulse may contain about 10^11^ hard X-ray photons, over 98% of which (at 12 kV) pass through a protein crystal without interaction. Of the remaining 2%, 84% are annihilated in the production of photoelectrons, 8% are scattered by the Compton process and 8% are Bragg scattered.

For the case of a 40 fs, 2 keV pulse with irradiance 10^17^ W cm^−2^ (which has been analysed in detail), 10% of the C atoms in a protein crystal absorb a photon and are ionized, a process that we might describe as primary or electronic damage. A cascade of photoelectrons and Auger electrons releases this energy, followed by a cascade of low-energy electrons caused by secondary impact or field ionizations taking place on a 10–100 fs timescale. Coulomb repulsion of the ions and an increase in electron temperature then cause displacement of both atoms and ions during the pulse, resulting in the secondary-damage process, which can be avoided by using sufficiently brief X-ray pulses. This heating leads to vaporization of the sample if the secondary electrons cannot escape, as the temperature rises. Higher beam energies produce weaker scattering, both Bragg and inelastic, with Compton scattering replacing the photoelectric effect that is dominant at lower energies (Attwood & Sakdinawat, 2016 ▸). For the single-particle (SP) mode, in the absence of Bragg diffraction, damage effects are manifested in a different way. Simulations by both molecular dynamics (Hau-Riege, 2012 ▸) and hydrodynamic codes (Caleman *et al.*, 2011 ▸) predict that 0.5 nm motions of the ions can occur in less than 100 fs, so that pulses as short as 10 fs may be required to achieve atomic resolution, a more demanding requirement than that for SFX, which benefits from Bragg scattering summed over the periodic­ally arranged molecules and a consequent ‘Bragg boost’: the squaring effect owing to coherent amplification mentioned previously. (This will, however, be modified by the ‘gating’ effect discussed below.) It is found that doses of up to a thousand times greater than the Garman–Henderson ‘safe dose’ can be used in SFX for similar resolution, if sufficiently brief XFEL pulses are used, which apply the dose at a much higher rate (Lomb *et al.*, 2011 ▸; Barty *et al.*, 2012 ▸). More specifically, if the ‘safe dose’ is about 30 MGy for cooled samples at synchrotrons (or 0.2 MGy at room temperature), then it is estimated to be about 700 MGy for an XFEL using 70 fs pulses [see Chapman *et al.* (2014 ▸) for a full discussion]. Recently, site-specific damage effects have been imaged in density maps around Fe metal clusters in ferredoxin using XFEL data (Nass *et al.*, 2015 ▸) and compared with synchrotron results. A submicrometre beam focus was used at maximum XFEL intensity, with beam energies above and below the Fe *K* edge for comparison. This work, and supporting simulations (Hau-Riege & Bennion, 2015 ▸), suggest that pulse durations of 20 fs or less may be needed to minimize some types of site-specific damage when using the smallest beam focus for highest intensity in single-particle (SP) imaging, particularly if heavy atoms, which produce a strong local shower of photoelectrons, are present.

**Figure 4:**
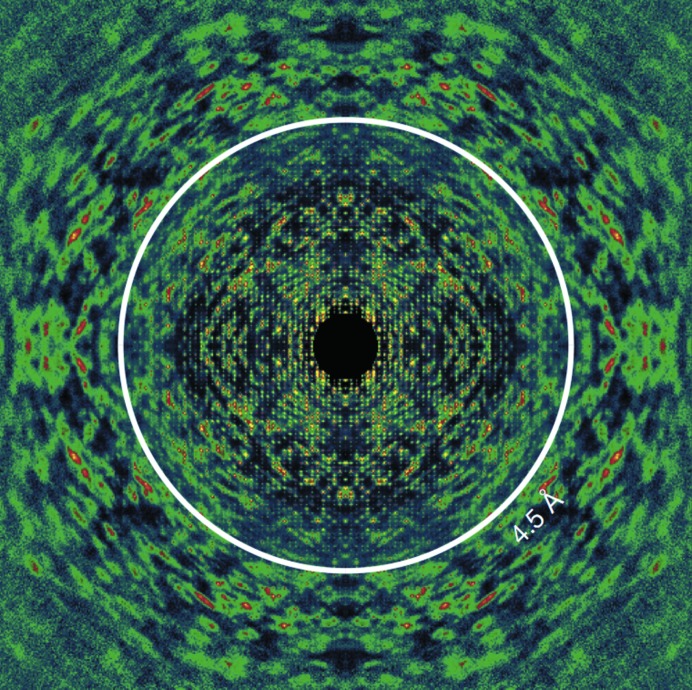
XFEL diffraction snapshot from photosystem II crystals. This is a section with [100] normal through the origin of a three-dimensional data set merged from thousands of microcrystals after indexing. The diffuse scattering, owing to static displacements of the molecules, is seen to extend well beyond the Bragg reflections and can be used both to increase the resolution and assist phasing (Ayyer *et al.*, 2016 ▸).

Spot-fading studies (Fig. 3 ▸) show how the disappearance of the outer Bragg reflections ‘gates’ the time-resolution of the process: the effective pulse duration which matters is the time taken for these spots to fade, destroying translational symmetry before the pulse ends, not the duration of the pulse (Barty *et al.*, 2012 ▸). For single particles, the onset of damage is more difficult to determine from the continuous distribution of scattering in the patterns and will need to be studied by the modeling of known structures once reliable high-resolution data have been obtained from monodisperse particles. At present, the resolution of three-dimensional reconstructions from SP data (about 10 nm) is not sufficient to observe these damage-limiting effects on resolution. The diffraction pattern shown in Fig. 4 ▸ (discussed below) contains information on both single molecules and crystallographic diffraction, and so might be used to resolve this issue, since the diffuse scattering shown is an incoherent sum of the intensity of scattering from the individual molecules in the crystal, unlike the Bragg beams, which are a coherent sum.

## 4. Serial crystallography at XFELs   

While the bulk of protein structure analysis can best be undertaken at synchrotrons, XFEL crystallography has been found to offer the following advantages.

(i) The reduction in radiation damage observed when using 10 fs pulses allows crystallography at room temperature without the need for cooling to avoid damage, and in a controlled chemical environment, from the smallest (for example submicrometre) crystals, from which useful data cannot readily be obtained at synchrotrons. This opens the way to the study of dynamics at room temperature. (X-ray crystallography until about 1990 was normally undertaken at room temperature, and this approach is still used in time-resolved work, but without the damage-amelioration benefit of the XFEL.) The sample temperature will depend on the type of sample delivery used, from room temperature for samples studied at atmospheric pressure on fixed-sample scanned arrays (Roedig *et al.*, 2016 ▸) to somewhat below room temperature when using a liquid jet with the X-ray beam positioned very near the nozzle. [The temperature decrease along a water jet, owing to evaporative cooling in vacuum, has been measured and calculated (see Weierstall *et al.*, 2008 ▸), resulting in the formation of ice balls.]
(ii) Showers of microcrystals are frequently observed during crystal-growth trials, yet it may take months or years to find the conditions required to grow crystals that are large enough for conventional crystallography. Time-consuming screening trials can be avoided by direct injection of these microcrystals in a liquid jet or a similar sample-delivery device. Since diffraction patterns have been obtained from nanocrystals of just a few dozen molecules on a side, research into the identification of ‘invisible’ protein nanocrystals that are too small to be detected by optical microscopy continues, using methods such as SONICC (Haupert & Simpson, 2011 ▸). Methods for growing the required microcrystals are under continuous development: these include growth in LCP (Liu *et al.*, 2013 ▸) and growth in living cells, with extraction from the cells or with the cells themselves injected into the XFEL beam (Gallat *et al.*, 2014 ▸). Crystals larger than a micrometre are preferred for LCP work in view of the LCP background. Micro-electron diffraction in the TEM has recently also been used for the study of protein nanocrystals. For the small-molecule amyloid crystals important for Alzheimer’s disease, the build-up of strain in the crystals limits the crystal size (Sawaya *et al.*, 2016 ▸).
(iii) The higher time resolution possible using an XFEL.
(iv) Noncyclic reactions can be studied (since each sample is destroyed), rather than requiring cyclic low-dose stroboscopic conditions on the same sample region.
(v) When using crystals of a few micrometres in size, the optical absorption length for pump lasers is comparable with the crystal dimensions, allowing saturated pumping.
(vi) For diffraction studies of microcrystals reacting with a substrate, as discussed in more detail below, diffusive mixing is possible, since the diffusion time of the substrate into the crystals is short (Schmidt, 2013 ▸; Wang *et al.*, 2014 ▸).
(vii) In several cases, the resolution appears to be better at XFELs than at synchrotrons for similar protein crystals; however, detailed tests of these claims and comparisons with full control of crystal quality, dose, temperature factors and beam diameter remain to be performed and will be difficult. The general trend seems to be that for microcrystals, radiation damage at synchrotrons results in lower resolution data than from an XFEL. For large crystals, synchrotron resolution may be better given sufficiently high-quality crystals.
(viii) Inner-shell X-ray absorption (Mitzner *et al.*, 2013 ▸; Kroll *et al.*, 2016 ▸) and emission (Kern *et al.*, 2015 ▸) spectra may be collected in synchrony with snapshot X-ray scattering from microcrystals, allowing the chemical and spin states of heavy atoms to be tracked in time through a chemical reaction, in correlation with density maps, using pump–probe or mixing experiments.

More than 100 structures determined using an XFEL have been deposited in the Protein Data Bank (PDB). Recent SFX examples include the GPCR angiotensin receptor (important for drugs which control hypertension) at 2.9 Å resolution (Zhang *et al.*, 2015 ▸), rhodopsin bound to arrestin (Kang *et al.*, 2015 ▸) and cytochrome *c* oxidase (Hirata *et al.*, 2014 ▸), while the structures of lysozyme, glucose isomerase, thaumatin and fatty acid-binding protein type 3 have also been reported at a resolution of better than 2 Å (Sugahara *et al.*, 2015 ▸), among many others, including the study of nitrite reductase by Fukuda *et al.* (2016 ▸). Serial crystallography itself has also been developed at synchrotrons (Stellato *et al.*, 2014 ▸; Standfuss & Spence, 2017 ▸), where the source brightness and detector speed may be sufficient to freeze crystal rotation during an exposure. SFX has also been undertaken at synchrotrons using viscous media to reduce the crystal rotation during a brief exposure (Botha *et al.*, 2015 ▸; Nogly *et al.*, 2015 ▸).

An important recent advance has been the realisation that in crystals for which the disorder consists solely of rigid-body displacements (without rotation) of proteins from the ideal lattice, the strong diffuse scattering seen between Bragg reflections in these snapshots is mostly just the single-particle diffraction pattern from one primitive unit cell, loosely described as the molecular transform. (Unlike the molecular transform, it does however fall to zero around the origin.) This is predicted by an extension (Ayyer *et al.*, 2016 ▸) of the Debye theory (Debye, 1913 ▸) of scattering from crystals with thermal motion and is shown in Fig. 4 ▸. Since this anisotropic scattering extends well beyond the Bragg reflections (and is not subject to thermal damping), this effect has now been used to extend the resolution of density maps of photosystem II from 0.45 to 0.35 nm (Ayyer *et al.*, 2016 ▸). Because it provides ‘oversampled’ data (intensity running between the Bragg spots), this continuous scattering can also assist in solving the phase problem and opens up the possibility of solving imperfect crystals. This approach might also be applied to liquid crystals, which possess orientational order but not translational symmetry; however, the power of crystallographic indexing would be lost for orientation determination.

## 5. Single-particle imaging: molecular machines   

Here, we briefly summarize progress towards the imaging of single particles (such as a virus) with one partlcle per XFEL shot, and address the unique insights which the time resolution and data volume of XFELs might provide for our understanding of the molecular machines of life. Single-particle methods are reviewed in Ekeberg (2015 ▸), Bostedt *et al.* (2016 ▸), Aquila *et al.* (2015 ▸) and Liu & Spence (2016 ▸). Related single-particle developments in Japan at the SACLA XFEL can be found in Kimura *et al.* (2014 ▸) (for live cell imaging) and Takayama *et al.* (2015 ▸) (for SP imaging of choloroplasts).

Increasingly, it has been realised that under physiological conditions proteins sample a large ensemble of conformations around the average structures given by crystallography, owing to the availability of thermal energy, and that this dynamic behavior, consisting of near-equilibrium fluctuations, is crucial to their function. Can XFEL single-particle imaging contribute to our understanding of these processes? This may be possible because of the very large amount of data that is obtainable at near-physiological conditions, for reasons that we now discuss. At higher temperatures, proteins switch rapidly between substates, but are inactive at the temperatures at which most crystal structures are determined. Controlling conditions include solvent chemistry (*e.g.* pH), pressure, local electric fields and ligand binding. Protein function is then the result of a complex interplay between thermal motions and their chemical and physical environment. The modern description of protein function is therefore based on a multi-dimensional energy landscape (Frauenfelder *et al.*, 2001 ▸; Wales, 2003 ▸) that defines the relative probabilities of the conformational states (the thermodynamics), the energy barriers between them (the kinetics) and the work cycle. This concept of a landscape was taken from the original Eyring transition-state theory in chemical dynamics. Historically, studies on myoglobin have led the way (see Fenimore *et al.*, 2004 ▸ and references therein). Molecular processes depend on alterations in rates and populations in an ensemble, such as enzymes facilitating reactions or changes in intracellular ion concentrations which trigger neurological processes. Very large rate increases can be achieved by very small changes in free energy (a few *kT*), so that the breakage of a few hydrogen bonds or van der Waals contacts in a protein containing thousands of such interactions can turn on a signaling cascade or catalyze a chemical reaction. Intrinsic protein dynamics only occur in this free-energy range of several *kT*. It has been suggested in one approach (Henzler-Wildman & Kern, 2007 ▸) that the conformational substates sampled by a protein and the pathways between them are not random, but rather a result of the evolutionary selection of states that are needed for protein function, and hence are ‘pre-formed’. Signal transduction, enzyme catalysis and protein–ligand interactions occur as a result of the binding of specific ligands to complementary pre-existing states of a protein and the consequent shifts in the equilibria. In this picture, the energy landscape is an essential, intrinsic property of a protein, encoded in its fold and central to its function: the ligand does not induce the formation of a new structure but instead selects from pre-existing structures, according to this school of thought. An alternative explanation assumes that conformations do provide an induced fit. A considerable complication arises from the fact that ligand binding modifies the energy landscape, so that the substrate must cross from the ligand-free landscape to the ligand-bound landscape.

Several types of protein dynamics may be distinguished, according to driving force, reversibility, speed, cyclic nature and thermodynamics. Certain molecular motors convert the chemical energy provided by ATP hydrolysis (12 kcal mol^−1^, 20 *kT* at 300 K or 0.52 eV per molecule) into mechanical motion. The molecular machines of life are otherwise mostly driven by thermal fluctuations (together with the input of chemical energy), operating on timescales longer than microseconds, as has been clear since the first studies on myoglobin in the early 1960s, where it was noted that structural fluctuations were needed to accommodate O_2_ diffusion (for a review, see Frauenfelder *et al.*, 2001 ▸). These molecular machines might be thought of as molecular structures which focus Brownian motion. In equilibrium, buffeted by the surrounding water molecules, these machines (such as the ribosome and kinesin), may be said to be idling. (Kinesin, which in equilibrium is equally likely to move to left or right on microtubules, moves only in one direction when provided with chemical energy.) Another type of system are the light-sensitive proteins, which respond to photon energies much larger than *kT*. Note that while time-sequence information is not needed to map out the energy landscape, it is needed to determine the way that the path adopted by a particular driven system is traversed. Relative energies for molecular conformations in a work cycle operating with energies around *kT* can be obtained from the ratio *n*_1_/*n*_2_ of the populations of two particular conformations in equilibrium, since this ratio is given by a Boltzmann exponential factor. In this way, if we assume that we need to observe only the *n*_1_ = 1 example of the most extreme highest energy conformation quenched from an ensemble, we may find that energy difference for any given total number *N* = *n*_1_ + *n*_2_ of reconstructed density maps from the ensemble (Dashti *et al.*, 2014 ▸). A recent example of the determination of an energy landscape for the ribosome using cryo-EM data can be found in Dashti *et al.* (2014 ▸). Thus, the much larger amount of data (larger *N*) obtainable in XFEL SP experiments (especially when using the new high-repetition rate machines) will give access to these much rarer, larger energy and larger conformational changes that are not seen in cryo-EM imaging and which may be important for physio­logical function. These may be rate-limiting (and go beyond the harmonic approximation commonly made in molecular-dynamics simulations or seen by crystallography). For future data sets obtainable from the European XFEL over a few days, with very large values of *N* (and *n*_1_ = 1), a simple estimate shows that these energy differences may exceed the energy available from ATP per molecule. These very large conformational changes would then be visible at a moderate resolution of, perhaps, 1 nm. In this way, one may go beyond the small conformational changes imposed by the study of proteins which can be crystallized, which can only provide a periodic average over all conformers in the crystal, and the limitations on particle size imposed in NMR studies of dynamics (Lewandowski *et al.*, 2015 ▸). Such a large high-energy conformational change must lie on the minimum-energy pathway important for enzyme function to be rate-limiting. Pump–probe SP studies would also have the important advantage of providing time-sequence information.

Since most biochemical reactions occur on a timescale of a microsecond or longer, the value of XFEL imaging on the femtosecond timescale (other than for reduction of damage) in biology has been questioned. However, in fact, as has been pointed out, all timescales are relevant, from the excited-state lifetimes of the initial electronic excitation (which may be very brief, or extended) onwards (Moffat, 2014 ▸). Enzymes, for example, rely on fluctuations that are much faster than the enzymatic constants. The binding of charged ligands can be electrostatically steered over very small diffusional distances, and therefore over very short times. Other reactions depend upon the formation of the correct cluster of ions with extremely short lifetimes. The crucial initial stages of light-driven processes such as human vision and photosynthesis clearly play out in the femtosecond regime.

It is useful here to distinguish chemical reaction *dynamics*, which are defined as molecular processes on the atomic scale (often involving electronic excitations) over very brief timescales (Levine, 2005 ▸), from the *kinetics* describing the appearance and decay of intermediate species over longer times by rate equations, leading eventually to a final state of thermodynamic *equilibrium*. All of these insights deepen our understanding of biochemistry, and improved time and spatial resolution can also provide more accurate refinement of the atomic potentials used in molecular-dynamics simulations.

The Single-Particle Initiative (SPI) program at LCLS consists of beamtime set aside by the LCLS Director for dedicated noncompetitive beamtimes over a multi-year period to systematically trace, identify and rectify the resolution-limiting factors in SP diffraction at LCLS. Steady progress has been made in reducing beamline background scattering and improving detector performance, resulting in a steady improvement in resolution. For soft X-ray data, where the scattering is stronger, data collected under different conditions at the LAMP chamber (at the AMO experimental station at LCLS) and merged and phased for three-dimensional reconstruction show about 10 nm resolution images of a virus capsid, while smaller amounts of data can be obtained showing much higher resolution in individual shots. The quality of the data collected in single-particle experiments also depends on accurate detector characterization, lateral jitter in beam position, the impact parameter for the hits, the amount of salts which may ‘plate out’ onto the surface of the particle (‘caking’; Kassemeyer *et al.*, 2012 ▸) and the X-ray background from stray (parasitic) scattering (Munke *et al.*, 2016 ▸). This background has been greatly reduced using a system of shadowing apertures, in particular a small aperture placed slightly downstream of the sample, which blocks upstream background sources, such as X-ray scattering from aperture edges and asperities (Awel *et al.*, 2017 ▸). Since the scattering from a dielectric sphere falls off as the inverse fourth power of the scattering angle, the limited dynamic range of current X-ray detectors is a serious problem. However, with continued progress it is reasonable to expect that 1 nm resolution or better will be achieved before long, with much larger amounts of SP data available soon from the new European XFEL.

## 6. Fast time-resolved serial crystallography   

The term ‘molecular movie’ has been widely used and misused in the literature. Leaving aside animations and the question of how direct the observations are (from modeling based on fast optical spectroscopy, from diffraction data, from imaging using lenses *etc.*), for our purposes it is important to distinguish between methods which involve ensemble averaging (for example by detecting Bragg diffraction from a crystal in which the molecules are undergoing a chemical reaction) and those which do not [such as cryo-EM and time-resolved single-particle imaging based on XFEL diffraction (TR-SP)]. A further important distinction can be made between ‘trapping’ (or quenching) experiments, in which molecules in thermal equilibrium are rapidly quenched and their images are then sorted by conformation, and pump–probe experiments, in which molecules are excited before having their snapshot taken after a controllable and measured delay. (In cryo-EM molecules can also be optically excited before freezing in a thin vitreous ice film.) The analysis and interpretation of movies made from ensemble-averaged data (for which Bragg diffraction can then provide the highest atomic resolution images) is discussed below: we shall describe these as ‘molecular movies’ or ‘movies’ for brevity.

Experiments on light-sensitive proteins use micrometre-sized protein crystals excited in a liquid jet upstream of the X-ray pulse where their snapshot is recorded. The time delay between excitation and X-ray interaction (which corresponds to one frame of a ‘molecular movie’) may be determined either (most accurately) by timing electronics (with pump illumination spatially extended along the flow) or by the flow time in the liquid stream (less accurately, but allowing longer delays). Thousands of snapshots are required (with the crystals in random orientations) for each delay (movie frame) to build up a three-dimensional diffraction data set. Steady improvements in SFX data-analysis algorithms (discussed below) beyond simple Monte Carlo averaging can now resolve the small changes of a few percent in structure-factor magnitudes owing to optical illumination of a micrometre-sized protein crystal, despite the scaling problems owing to the continuous variation in crystal size and orientation while working with partial reflections. (The ability to detect the even smaller changes in structure-factor magnitudes used for SAD phasing of XFEL data is an even more severe test of data quality.) The first TR-SFX results were obtained by Aquila *et al.* (2012 ▸) for photosystem I–ferredoxin. More recent examples, incorporating many advances in instrumentation and the improvement over synchrotrons in time resolution (up to 1000 times) when using an XFEL, can be found in Barends *et al.* (2015 ▸) for myoglobin, Kupitz *et al.* (2014 ▸), Young *et al.* (2016 ▸) and Suga *et al.* (2017 ▸) for photosystem II, Nango *et al.* (2016 ▸) for bacteriorhodopsin, and Tenboer *et al.* (2014 ▸) and Pande *et al.* (2016 ▸) for photoactive yellow protein (PYP).

In a protein crystal excited by a femtosecond optical pulse, in which it has been established from prior spectroscopic studies that there are two reaction paths around a work cycle (such as the photoactive yellow protein discussed below), molecules in different unit cells have certain probabilities of either not being excited at all or initiating a reaction on either path. (Further branching may also be a possibility.) Each path, described by chemical rate equations, will produce different intermediate species with different rate constants. Measurement of lattice constants, temperature factors and overall resolution provide assurance that the crystal remains intact during the cycle, and that the outer envelope and contact points between molecules in different unit cells are little affected. (Destruction of the crystal by the photoelectron cascade in each shot comes later.) The observables, from a stream of microcrystals, are the Bragg reflections, which after phasing provide a periodic spatial average of the electron density from the average of all illuminated crystals at one time point (the pump–probe delay). The method therefore requires accurate knowledge of the un-illuminated (dark) ground (or final) state structure from prior crystallography at the highest resolution. Methods such as singular value decomposition (or modeling using molecular dynamics) can then be used to separate the time-resolved charge densities along each path from the Bragg data. From this, the amounts of intermediate species which come and go during the reaction cycle can be extracted based on the rate equations describing the reaction kinetics.

**Figure 5:**
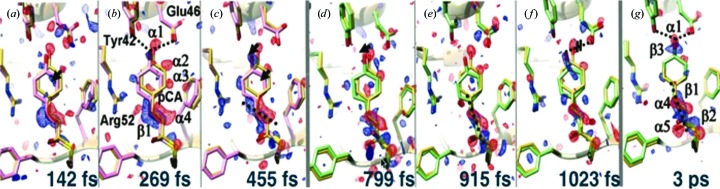
*Trans*-to-*cis* isomerization in PYP. Weighted difference electron-density maps are shown in red (−3σ) and blue (3σ). The reference structure is shown in yellow, structures before the transition (but still in the electronic excited state) are shown in pink and the structure after transition in the ground state is shown in green. Important negative features are denoted α and positive features are denoted β. Pronounced changes are highlighted with arrows. (*a*, *b*, *c*) Representative time delay before transition. Dashed line: direction of the C2=C3 double bond, feature β1. Dotted lines: hydrogen bonds of the ring hydroxyl to Glu46 and Tyr42. Chromophore configuration from 100 to 400 fs pump–probe delay. (*d*) Chromophore configuration at 799 fs after transition. (*e*, *f*) Chromophore configuration at longer times from 800–1200 fs. (*g*) 3 ps chromophore configuration; the dashed line shows the direction of β (Pande *et al.*, 2016 ▸).

Fig. 5 ▸ shows the work of Pande *et al.* (2016 ▸), who achieved a 200 fs time resolution over a 3 ps range in their TR-SFX study of photoactive yellow protein, which was sufficient to provide several frames of a 0.16 nm resolution movie of the *trans*/*cis* isomerization reaction which results from photon absorption in this light-sensitive protein. The mechanism is the same as that which occurs in the first event in human vision (in a different protein matrix), when photons strike rhodopsin at the retina. This involves a conical intersection [a degeneracy in nuclear coordinates for the excited and ground states (Schoenlein *et al.*, 1991 ▸)]. This TR-SFX experiment was performed using the GDVN pump–probe liquid-injection system shown in Fig. 1 ▸. Laser illumination (simulating the effect of sunlight on a plant or organism) causes a small change in structure factors, which can be phased by the molecular-replacement method to produce a difference density map between the bright (optically pumped) and dark states for each time delay. This project followed earlier work on the same system over a longer time interval using the same method at a lower time resolution (Tenboer *et al.*, 2014 ▸).

It is clear that much more accurate results could be obtained if the Laue method, as previously adopted for this work, could be used (see, for example, Schotte *et al.*, 2003 ▸). Here, a wide energy spread in the beam is used to provide a ‘thicker’ Ewald sphere which spans the full angular profile of the Bragg peak, allowing each snapshot to record full reflections (for a single projection) at each time point and eliminating the need to scale Bragg peaks between different sized crystals of different partiality. The required large energy spread d*E*/*E* is, however, not normally possible using a monochromatic X-ray laser. (For the LCLS, d*E*/*E* ≃ 0.1%; for a synchrotron, d*E*/*E* ≃ 0.02% is common.) Moffat (2014 ▸) finds that to provide angle-integrated intensities from a crystal with mosaic disorder dφ = 10^−2^ and Bragg angle β, one requires d*E*/*E* > φcotβ. For a high-angle reflection with β = 0.3 rad, this requires d*E* = 260 eV at *E* = 8 kV, or less for more perfect crystals, and more for low-angle reflections. The suggestion has been made that the submicrometre-sized crystals sometimes used for SFX are more perfect, since their size is likely to be smaller than one mosaic block. However, this model may not apply to many proteins, the defect structures of which are not well known (Snell *et al.*, 2003 ▸). The use of a ‘chirped’ beam (which changes energy during the pulse) and the use of ‘two-color’ methods have also been proposed. Here, the XFEL generates pairs of pulses with a tunable femtosecond-scale delay at slightly different wavelengths. For an analysis of errors in SFX using two colors, see Li *et al.* (2015 ▸).

A promising approach is the use of genetic engineering to create light-sensitive protein domains within a system of interest, known as opto-genetics. If microcrystals can be grown, this would provide a general method of studying protein dynamics (Moffat, 2014 ▸).

New approaches to XFEL time-resolved diffraction have been reviewed in Spence (2014 ▸), including the use of atto­second pulses of duration Δ*t*. Here, the unavoidable broadening of the energy spread Δ*E* (eV) = 4.14/Δ*t* (fs) in a band-limited beam could provide just the conditions needed for Laue diffraction: 14-attosecond pulses would provide 3% bandwidth at 10 kV. In addition, the temporal coherence allows Bragg beams from different reflections, excited at different wavelengths but diffracted in the same direction, to interfere briefly (for the duration of the beating period), contributing to solution of the phase problem by providing three-phase invariants (Spence, 2014 ▸).

## 7. Slow time-resolved serial crystallography: mixing jets   

Solution-scattering experiments at synchrotrons can provide diffraction from a mixture of solutions during a chemical reaction (Van Slyke *et al.*, 2014 ▸). The reaction may be triggered in some way, or result from mixing, prior to chemical reaction of the species. The mixing time determines the time resolution of the method. Reactions can be triggered by the photoelectrons generated by the X-ray beam itself, as in the cases of cytochrome P450 (Schlichting *et al.*, 2000 ▸) and horseradish peroxidase (Berglund *et al.*, 2002 ▸). The high brightness of modern synchrotrons and fast detector speeds have therefore recently enabled serial crystallography methods to provide ‘molecular movies’ of enzyme mechanisms triggered by the beam, in which the radiation dose is kept well below the Garman–Henderson ‘safe dose’ and resolution loss during the reaction is minimal (Horrell *et al.*, 2016 ▸). Using an XFEL in serial crystallography mode, it becomes possible to use micrometre-sized crystals, so that rapid diffusive mixing into the crystals becomes possible with such small crystals, and the crystals can provide atomic resolution data. [The diffusion time for glucose into a 1 µm crystal of lysozyme, for example, is about 20 ms (Schmidt, 2013 ▸).] Furthermore, radiation damage can almost be eliminated, thus disentangling the effects of damage from the chemical reaction. Most importantly, the chemical reaction can then be imaged by snapshot X-ray diffraction at room temperature under near-physio­logical conditions, where the correct thermal energy is available to take part (with other driving forces) in driving these reactions. A description of the first double-focusing GDVN ‘mixing jet’ for XFEL sample delivery is given in Wang *et al.* (2014 ▸) and a more recent design can be found in Calvey *et al.* (2016 ▸); these have now been used successfully at LCLS.

**Figure 6:**
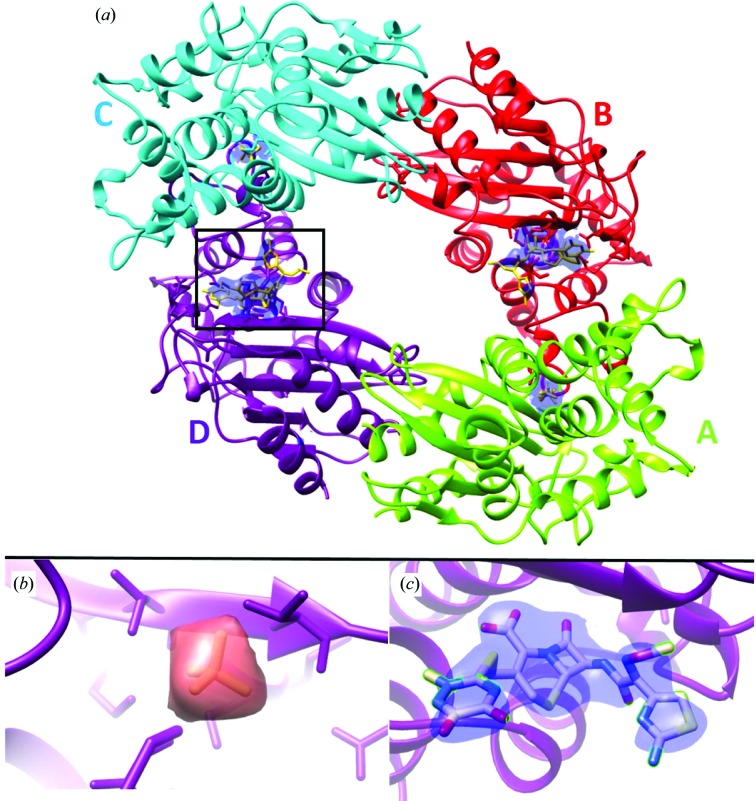
Electron density in the catalytic cleft of BlaC. (*a*) Refined model of the entire tetramer (σ = 1.1) in the asymmetric unit after mixing. The electron density (2*F*_o_ − *F*_c_) is shown in blue in the binding pockets. Subunits *A* and *C* contain phosphate; subunits *B* and *D* have a bound ceftriaxone, with that in subunit *D* being bound slightly more strongly. (*b*) Enlarged section of the apo (red electron density) subunit *D* binding pocket showing electron density for phosphate. (*c*) Enlarged section of the mixed (blue electron density) subunit *D* binding pocket showing electron density for ceftriaxone. (*b*) and (*c*) show slightly different views of the same subunit-binding pocket; however, there are minimal changes to the ligand-binding sphere. From Kupitz *et al.* (2016 ▸).

Fig. 6 ▸ shows the results of such a time-resolved mixing experiment at an XFEL (Kupitz *et al.*, 2016 ▸). Here, the reaction between the enzyme β-lactamase (BlaC) and a small drug molecule, ceftriaxone (boxed in the figure), has run to completion using solutions of the drug molecule and enzyme microcrystals which were mixed before delivery to the GDVN jet. The density map shows the drug bound into the enzyme ring at two locations. A four-frame movie of the drug molecule during binding is under development. A second example can be found in Stagno *et al.* (2017 ▸) for the adenine riboswitch RNA aptamer, where a 10 s delay after mixing captures the structure of an intermediate phase.

We can now foresee a much wider range of methods being used to trigger reactions for imaging dynamics at XFELs in the near future. These might include terahertz pumping (of the hydration shell around proteins, which couples *via* a dipole interaction), temperature-jump and temperature-equilibrium measurements, and particularly caged-molecule release experiments (Schlichting, 2000 ▸), including pH changes driven by optical pumping of proton-release cages (see, for examle, Lommel *et al.*, 2013 ▸) and other photolabile compounds.

## 8. Fast solution scattering and angular correlations   

We will refer to wide-angle X-ray scattering (WAXS) using an XFEL as ‘fast solution scattering’ (FSS). Apart from reduced radiation damage, the XFEL offers the advantage of improved time resolution. As a result, we have seen remarkable studies of the phase transitions in water at low temperature (Nilsson *et al.*, 2016 ▸) and of photosensitive protein molecules by time-resolved pump–probe XFEL solution scattering (Arnlund *et al.*, 2014 ▸; Kim *et al.*, 2015 ▸). In the study by Arnlund and coworkers of the *Blastochloris viridis* reaction center, 500 fs time resolution and about 0.4 nm spatial resolution were obtained in the difference maps between the optically pumped and dark states, allowing a molecular movie to be obtained following photon excitation. (Prior crystallography had provided an accurate dark-state structure, allowing extensive modeling by molecular dynamics.) The time-dependent diffraction provided details of the ‘quake’ mechanism responsible for dissipating energy, which prevents unfolding of the protein following absorption of the large photon energy (2.5 eV >> *kT*). Time constants were obtained for both the initial quake motion (7 ps) at lower scattering angles and the later high-*q* heating process (14 ps). The epicenter of the ‘quake’ (Ansari *et al.*, 1985 ▸) was seen to occur at the chlorophyll cofactors. In a similar way, Levantino *et al.* (2015 ▸) have published TR-FSS studies of carbonmonoxy myoglobin, using LCLS data to observe light-induced structural rearrangement following photolysis of the heme iron–CO bond and the resulting ‘quake’ motions. They see damped oscillations with a 3.6 ps time period. For inorganic reactions, Kim *et al.* (2015 ▸) have published remarkable FSS observation of interatomic bond formation in the gold trimer complex [Au(CN)_2_^−^]_3_. The reaction is optically triggered between Au atoms in close proximity, avoiding time delays owing to diffusion, at sub-angstrom resolution and 200 fs time resolution.

It has been pointed out that solution scattering from molecules frozen in time or space should be anisotropic, containing speckles (additional to the effect of coherent interparticle scattering), unlike synchrotron WAXS data which are isotropic because the molecules rotate during exposure. This type of scattering has been termed fluctuation X-ray scattering (FXS; Kam, 1977 ▸) or correlated fluctuation scattering. Furthermore, a method exists for extracting the electron-density map (image) of one particle using this anisotropic scattering with many identical, randomly oriented particles per shot in solution (Kam, 1977 ▸). Clearly, such two-dimensional FSS patterns contain more information than the one-dimensional data to which WAXS patterns are reduced, facilitating inversion to three-dimensional models. The FXS patterns nevertheless lack the full information needed for three-dimensional reconstruction (Elser, 2011 ▸). A tutorial review of the theory of Kam and its history can be found in Kirian (2012 ▸). The concept can be understood in the simple case of two-dimensional identical objects lying flat on a plane normal to the beam, which differ only by random rotations about the beam direction. The two-dimensional angular correlation function (ACF) for each particle will then be independent of its orientation, allowing them to be added together. (The ACF is the autocorrelation function of the diffracted intensity taken around each resolution ring in the diffraction pattern.) With many particles per shot, it can be shown (Kirian, 2012 ▸) that this anisotropic ACF formed from diffraction patterns with many particles per shot consists of the one-particle ACF added to a conventional WAX background, which can be subtracted because it is isotropic. In principle, the resulting ACF can then be inverted to become a real-space image by phasing and Fourier transforming the data twice: once to convert the ACF to the diffracted intensity and a second phasing and transform to give the real-space image (Saldin *et al.*, 2011 ▸).

This anisotropy in FXS has been observed in X-ray scattering from colloidal glasses (Wochner *et al.*, 2009 ▸) and from randomly oriented gold nanorods lying flat on a membrane (Saldin *et al.*, 2011 ▸). These data were inverted using the Kam theory to provide an experimental image of a typical nanorod. For proteins in solution, the anisotropy in XFEL FXS data (with a recording time much shorter than the rotational diffusion time of the molecules) is usually swamped by other experimental artefacts that cause anisotropy. Success has, however, been achieved using two-dimensional lithographed structures (Pedrini *et al.*, 2013 ▸) at low resolution and from data in the PDB for a ligand-gated ion channel (pLGIC) using an important new development of the Kam approach (Donatelli *et al.*, 2015 ▸) which provides inversion to an image with a single phasing step. A significant theoretical finding is that the results of this method are independent of the number of particles per shot (Kirian *et al.*, 2011 ▸); however, experimental resolution (in the absence of modeling) appears to be better using the single-particle method (with one particle per shot). It is difficult to improve on the SP mode with a direct hit and the beam diameter matched to the particle diameter; however, experimental impact parameters (the distance between the center of the particle and of the beam) are rarely zero and hit rates are low (*e.g.* 1% or less), whereas FXS (many particles per shot) has a 100% hit rate. Thus, the optimum number of particles per shot (and analysis method) remains to be determined for real experimental conditions, including background scattering and variations in impact parameter. The Kam angular correlation method should be particularly powerful for known structures when detecting differences between ground-state and excited-state structures in solution scattering, where many sources of error are eliminated in these difference measurements (Pande *et al.*, 2014 ▸). Experimental FXS results showing strong anisotropy have been obtained from polymer dumbbells in solution at LCLS, where the Kam angular correlation method was used to reconstruct an image of one dumbbell (Starodub *et al.*, 2012 ▸). Here, the difference in sample density from the host solution is small, as for a protein. This paper, together with Kirian (2012 ▸), provides an excellent introduction to this promising approach to single-particle imaging.

## 9. Data analysis   

### 9.1. Serial femtosecond crystallography   

**Figure 7:**
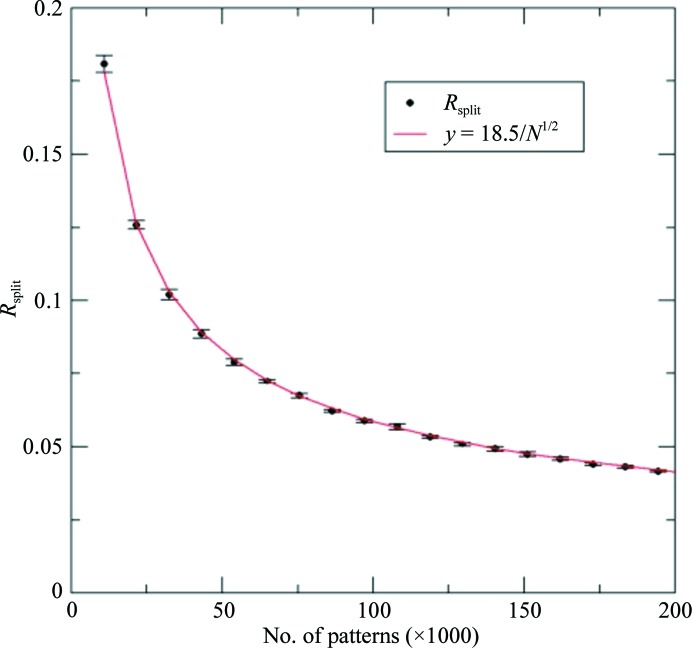
The experimental reduction in scattering-factor error measurement (*R*_split_) with increasing number *N* of diffraction patterns follows a Poisson error law *R*_split_ = *k*/*N* ^1/2^. For this SFX analysis of the photosystem II complex (PDB entry 3wu2) *k* = 18.5. Progress in SFX algorithm development, partial reflection analysis and scaling is measured by the reduction in *k* in recent years.

SFX diffraction patterns have required new algorithms for data analysis, while the high spatial coherence has provided new opportunities for solving the phase problem. During data acquisition, software (see, for example, Barty *et al.*, 2014 ▸) is used to discard blank shots and to identify good hits containing an indexable number of Bragg spots, to correct detector artifacts, to subtract background and to generate a virtual powder pattern (the sum of all good patterns showing Debye–Scherrer rings) for a quick indication of data quality and resolution, to possibly assist with indexing, to generate statistics on hit rate and resolution, and to convert the cleaned output to a standard file format such as HDF5. The autoindexing of these snapshot data remains an active field of research, which is complicated by the fact the Bragg reflections are ‘partial’ reflections. Since crystals (destroyed by each shot) cannot be rocked through the Bragg condition to provide the angular integration needed for a full estimate of a structure factor (so that goniometers are rarely used), new algorithms which address the scaling issues created by beam-intensity fluctuations, variations in crystal size and the precise determination of crystal orientation from diffraction-pattern intensities and geometry had to be developed. Expressions for XFEL diffraction by protein nanocrystals were first derived from first principles by Kirian *et al.* (2010 ▸), since when several software packages for SFX analysis have been developed and made available, such as *CrystFEL* [White *et al.* (2016 ▸); see also Ginn *et al.* (2016 ▸) for the *cppxfel* package]. New features of SFX patterns from the smallest crystals include the interference fringes between Bragg spots, which correspond to the ‘shape transform’ or Fourier transform of the external shape of the nanocrystal. This function, laid down around every Bragg peak, has an angular width in reciprocal space of approximately λ/*D* for a crystal of width *D*, and makes one contribution to mosaicity for larger crystals. A scattering vector must first be assigned to every Bragg spot, and this then provides the rotation matrix, which must be determined for each shot between the crystal and the laboratory frame. Indexing has mostly been achieved using standard crystallo­graphy software (for example *MOSFLM*; Winn *et al.*, 2011 ▸), allowing the data from many microcrystals to be merged into a three-dimensional diffraction volume. However, newer algorithms developed specially for SFX data, and tested on experimental data, can now index patterns using fewer spots (about five; Li *et al.*, 2017 ▸). These steady improvements in algorithms, which require fewer spots, allow the use of a larger fraction of the total amount of data collected, and so reduce the amount of protein and beamtime required. They also provide auto-indexing for the sparse data from crystals with small unit cells. Indexing ambiguities, which arise when the point-group symmetry of the molecule is lower than that of the lattice, can be resolved using the expectation maximization and compression (EMC) method (Liu & Spence, 2014 ▸) or correlation coefficients and a clustering procedure (Brehm & Diederichs, 2014 ▸; see also Kabsch, 2014 ▸). This ambiguity means, for example, that data from two successive microcrystals could be mistakenly merged in merohedral twin-related orientations if indexing were based on the geometry of the Bravais lattice alone. A simplified version of this algorithm is implemented in *CrystFEL*. Initially, full reflections were obtained using a Monte Carlo approach, which relies on recording and merging randomly oriented crystals whose orientations span and adequately sample the rocking curve for every Bragg reflection. The resulting error in structure-factor measurements can be estimated from the spread between the Bragg intensities of even (*I*_even_) and odd-numbered (*I*_odd_) diffraction patterns (Boutet *et al.*, 2012 ▸), 
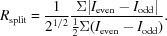
This falls off inversely as the square root of the number of patterns (with proportionality constant *k*) as errors are added in quadrature owing to variations in crystal size, orientation and a combination of impact parameter (the distance between the center of the X-ray beam and the center of the sample) and shot-to-shot variations in beam intensity, as shown in Fig. 7 ▸. This proportionality constant *k* has declined dramatically over the past six years as algorithms have improved and the sources of error (especially those associated with detector metrology, crystal size scaling and beam bandwidth) have been estimated or tracked down and reduced. Nevertheless, this Poisson scaling does mean that 100 times more data are needed to add one significant figure. The serial crystallography method, which avoids the use of a goniometer at pre-set measured orientations, amounts to ‘shooting first, and asking questions later’, as Rossman has commented. Much research has focused on the very difficult measurement of partiality or ‘post-refinement’ (Bolotovsky *et al.*, 1998 ▸). The fraction of a full reflection which is intercepted by the Ewald sphere and the precise deviation of each reflection from the exact Bragg condition defines partiality, as described in White *et al.* (2016 ▸), Uervirojnangkoorn *et al.* (2015 ▸), Kabsch (2014 ▸) and Sauter (2015 ▸). This is complicated by the fact that for a given range of energies, the ‘thickened’ Ewald sphere spans a wider range at high angle than at low angle. A significant advance has been the method of Ginn *et al.* (2015 ▸), which has provided 0.175 nm resolution structures from a few thousand protein microcrystals. A histogram showing the number of reflections predicted as a function of X-ray wavelength is used to refine the orientation matrix until a sharp peak is found in the histogram, which gives the beam-energy spread. Partiality is based on a model angular profile for the Bragg peak and spot locations are refined. This field of algorithm development for SFX data, including the iterative refinement of experimental parameters (particularly including the wavelength distribution in each X-ray pulse, variations in crystal size and diffraction conditions, and modeling of the Bragg profile), remains an active area of research which is producing large payoffs by reducing the amount of beamtime and protein needed to obtain an accurate structure. Additional experimental parameters might include variations in sample-to-detector distance, background owing to stray X-ray scattering, readout noise and pixel saturation. A comparison of XFEL and synchrotron data from lysozyme crystals has been given by Boutet *et al.* (2012 ▸).

The phasing of SFX data has been achieved mainly by the molecular-replacement (MR) method (Rupp, 2010 ▸), which uses a protein with similar sequence and fold in the PDB as a model structure. The single-wavelength anomalous diffraction (SAD) method has been successfully applied to XFEL data (Barends *et al.*, 2014 ▸; Nass *et al.*, 2016 ▸), as have isomorphous replacement (Yamashita *et al.*, 2015 ▸) and native SAD phasing using sulfur and chlorine (Nakane *et al.*, 2015 ▸; Batyuk *et al.*, 2016 ▸). This demonstrates the increasing accuracy of SFX data analysis. New *de novo* approaches for experimental phase measurement include measurement of the intensity dependence of scattering factors from heavy atoms (including sulfur), the ionization of which saturates following multiple *K*-shell/Auger ionization cascades, resulting in ‘hollow’ atoms. By sorting the data according to pulse intensity, an analysis similar to SAD or isomorphous replacement may then be applied (Son *et al.*, 2011 ▸). Finally, the interference fringes between Bragg reflections in the smallest crystals provide the ‘oversampling’ needed to solve the phase problem. For a nanocrystal immersed in a wide coherent beam, one finds (*N* − 2) interference fringes for a crystal containing *N* planes normal to the direction **g** running between the Bragg reflections in direction **g**. This is akin to the (*N* − 2) subsidiary maxima seen between the principal maxima in the optical transmission diffraction pattern from a grating of *N* slits. These fringes, running in several directions, therefore give the size of the crystal and may be used to solve the phase problem (Spence *et al.*, 2011 ▸). For an experimental demonstration of this approach, and additional references, see Kirian, Bean *et al.* (2015 ▸). In addition, for a diffraction-limited coherent beam of nanometre dimensions, the situation is analogous to that in the fully coherent scanning transmission electron microscope (STEM; Spence, 2013 ▸). If the beam-divergence angle is larger than the Bragg angle, these coherent diffraction orders overlap at the detector, producing interference fringes which depend on the absolute position of the beam with respect to the crystal lattice, and may be analysed according to the theory of ptychography for hard X-rays (Spence *et al.*, 2014 ▸).

### 9.2. Single particles   

For SP (single-particle) data analysis, with one particle, such as a virus, per shot, the methods of coherent diffractive imaging (CDI) have been adapted for XFEL data, including the hybrid input–output (HIO) algorithm (Fienup, 1982 ▸) and its variants [see Marchesini (2007 ▸) and Spence (2017*c* ▸) for reviews and Millane & Lo (2013 ▸) for a review of related iterative phasing methods in crystallography and the important constraint ratio concept]. Unlike the CXDI problem, the orientational relationship between successive diffraction patterns must first be determined using randomly oriented particles of unknown structure (and requiring a certain minimum number of detected photons), the accuracy of which may limit resolution, prior to solution of the phase problem. Approaches to these problems include the *GIPRAL* algorithm (Kassemeyer *et al.*, 2013 ▸), manifold embedding (Yoon *et al.*, 2011 ▸) and the expectation maximization and compression (EMC) algorithm (Loh & Elser, 2009 ▸), as widely used in cryo-EM, which has been applied to SP XFEL data (see Ekeberg, 2015 ▸ and references therein). Additional approaches are discussed in Kodama & Nakasako (2011 ▸), who apply methods similar to those used in cryo-EM to identify the water jacket in real space, requiring very high resolution data (and hence a very flat Ewald sphere), and in Sekiguchi *et al.* (2014 ▸), who describe the *SITENNO* software package for data collection, merging and phasing of single-particle data. Sekiguchi *et al.* (2016 ▸) describe a data-analysis scheme (*ASURA*) for assessing the accuracy of the retrieved density maps based on principal-component analysis. Takayama *et al.* (2015 ▸) describe a method for improving resolution by a factor of two and of phasing the data by adding dispersed colloidal gold particles near these fixed samples to generate a strong reference wave.

To fix ideas, the example of coherent hard X-ray scattering from a dielectric sphere, obtainable in closed form, is given in Starodub *et al.* (2008 ▸). Here, it is seen that the *q* ^−4^ fall-off with scattering angle leads to the problem in coherent diffractive imaging of having to simultaneously record strong intensity at low angles and much weaker intensities at higher angles, with the range of intensities often exceeding the dynamic range of the detector.

It may seem that for merging of thousands of diffraction patterns from similar randomly oriented single particles (such as a virus), the same methods as used in the cryo-electron microscopy (cryo-EM) community could be used. Here, noisy low-dose projection images of many copies of a particle, lying in many random orientations, are recorded within a single field of view, and must be merged to produce a three-dimensional image (Spence, 2013 ▸). However, XFEL diffraction patterns also require solution of the phase problem and, unlike real-space cryo-EM images, there is no requirement for correction of electron lens aberrations, while an enantiomorphous ambiguity arises from the Friedel symmetry of low-resolution diffraction patterns, which is not present for real-space images. In addition, diffraction patterns have an origin, unlike images, and the background owing to ice in cryo-EM images must be treated differently from the background in an X-ray diffraction pattern owing to diffraction from a water jacket surrounding the particle. Building on previous work on iterative phasing of continuous diffraction patterns, two main approaches have been developed for the reconstruction of a three-dimensional image (density map) from many randomly oriented snapshot single-particle X-ray diffraction patterns and for dealing with the associated problems of particle inhomogeneity. We will give here only a very brief outline of the general principles of these methods, focusing on key issues.

**Figure 8:**
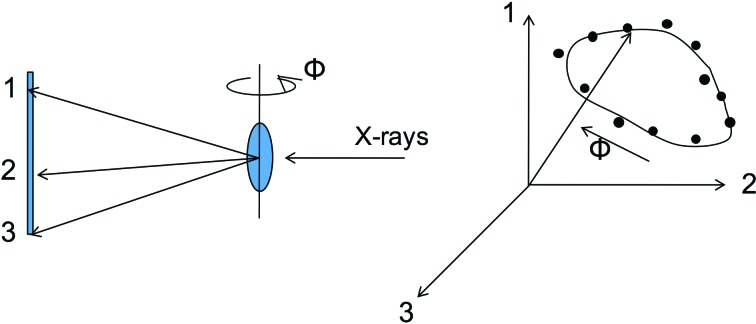
Simplified manifold embedding approach for a sample which can rotate only about one axis and a three-pixel detector. A vector in this three-dimensional space represents a diffraction pattern, each axis is a pixel and each coordinate value is an intensity for that pixel. Rotation of the molecule causes the vector to trace out a loop as the particle returns to its original orientation, while neighboring points on the loop represent similar diffraction patterns with small vectors χ (the least-squares difference, Euclidean metric) between their ends. Patterns recorded from molecules in random orientations can then be sequenced for a movie by identifying the loop path.

The manifold embedding approach (Yoon *et al.*, 2011 ▸) is illustrated in Fig. 8 ▸, simplified for the case of a three-pixel (*x*, *y*, *z*) detector and single-axis rotation of a particle in order to illustrate the principle of the method. With this simplification, a snapshot diffraction pattern can be represented as a three-dimensional vector, with each component representing the scattered intensity value at a pixel. These vectors (the diffraction snapshots) arrive in a random time sequence. However, the rotation of a particle traces out a loop (a one-dimensional manifold) in this three-dimensional space of intensities. Determining this manifold allows one to assign an orientation to each snapshot since, although the vectors arrive in a random sequence and position, they build up a loop which finally reveals their sequence and nearest neighbors. In general, the detector has *N* pixels and particle rotation about three axes generates a three-dimensional manifold in the *N*-dimensional Hilbert space of pixel intensities. The manifold is seen to be parameterized by a three-dimensional latent space defined by the three Euler angles defining the particle orientation. Many practical difficulties arise, including the transformation from angular increment to coordinate change in *N* dimensions, and the effects of noise and conformational changes. In the simplest case, a second conformation would define a second distinct loop; however, the effects of noise thicken the manifolds so that they may overlap. The key issue of distinguishing changes in particle orientation from conformational changes (essential in order to make a three-dimensional ‘molecular movie’) is resolved using the fact that the operations associated with conformational change commute, while those associated with the rotation group do not. Conformational changes alter the internal structure of a particle, unlike rotations. An important feature of this approach is that all of the data are used for all of the analysis, rather than selecting subclasses (for example of orientation or conformation) for successive analysis. However, even in the absence of noise, a minimum number of scattered counts is needed to identify a particular orientation, which is proportional to the number of distinct orientations sought. The computational demands of this approach are considerable and set the limit on the size of the largest molecule which can be analyzed.

A second approach is based on the principle of expectation maximization and compression (EMC; Loh & Elser, 2009 ▸; Sigworth, 1998 ▸; Dempster *et al.*, 1977 ▸). The method is most simply explained in two dimensions for the case of a set of noisy two-dimensional pictures *I*(*k*) of the same nonsymmetric object, which are known to lie in any one of four orientations *i* = 1, 4 differing by a 90° rotation about their normal. Here, *k* is the image index and extends over the *N* × *N* pixels of the pictures. A model is first assumed, which may consist of random values, and is generated in each of the four orientations *i* (expansion). Assuming Poisson noise, the probability *P*_*i*_(*k*) is calculated that an experimental image *I*(*k*) came from each model in orientation *i*. To avoid the occurrence of extremely small numbers in the first iteration, these probabilities are normalized to unity. The process is repeated (maximization) for each image, giving a set of coefficients *P*_*i*_(*k*). Four new models are then formed from the weighted sum *M*(*i*) = 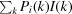. Since the four initial orientation-generating operations applied to the model are known, it is then possible to return the four new models to the same orientation, average them and use their average as a new estimate of the model. Iterations then continue from the first step. An experimental demonstration of the method using low-resolution two-dimensional X-ray shadow images has been demonstrated using as few as 2.5 photons per image (Ayyer *et al.*, 2014 ▸).

In both of these methods, solution of the noncrystallographic phase problem (reviewed in Spence, 2017*b* ▸) may be integrated with the problem of orientation determination.

**Figure 9:**
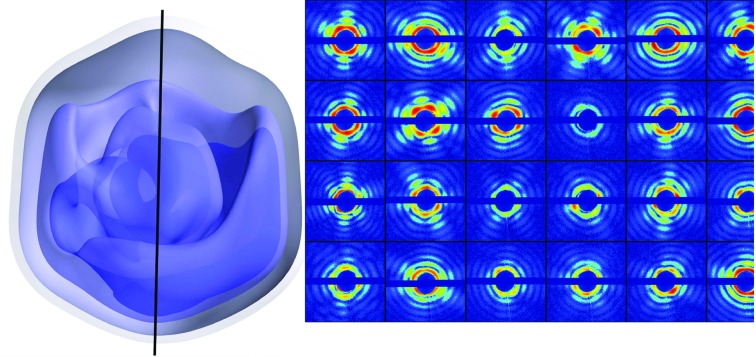
Three-dimensional reconstruction (left) of *Mimivirus* (450 nm diameter capsid) density at 125 nm resolution obtained using the *HAWK* software (EMC algorithm) from 198 single-shot diffraction patterns (right) obtained at LCLS [AMO, pnCCD detector, 70 fs pulses, 1.2 × 10^12^ photons per pulse (0.24 mJ), 1.2 keV X-rays].

Particle inhomogeneity (which increases with particle size) is the most important problem for single-particle XFEL imaging and may be solved in principle by the ability of the above methods to distinguish conformations if sufficient high-quality data are available. A method for obtaining a three-dimensional reconstruction from a single shot is described in Schmidt *et al.* (2008 ▸), using multiple incident beams split off by a beamsplitter. Several authors have pointed out that the curvature of the Ewald sphere provides limited three-dimensional information from a single shot. Bergh *et al.* (2008 ▸) describe other possibilities for extracting three-dimensional information from a single shot, such as Laue diffraction using harmonics, coherent convergent beam diffraction and multiple-pinhole Fourier transform holography. Fig. 9 ▸ shows the diffraction patterns (one particle per shot) obtained from *Mimivirus* particles, and the reconstructed three-dimensional image of the virus obtained using the EMC algorithm (Ekeberg *et al.*, 2015 ▸).

A database for SFX and SP data has been established, CXIDB (http://cxidb.org/index.html), where published data can be found and used to evaluate new algorithms. This site also makes available the *HAWK* program for EMC analysis of XFEL SP data.

## 10. Outlook   

As the focus of research in molecular biology moves from structure to dynamics as a result of more powerful computers for simulation and the invention of many new imaging techniques and spectroscopies, from NMR to laser tweezers, trapping experiments in cryo-EM and super-resolution optical microscopy, the XFEL has appeared on the scene at a propitious moment. For light-sensitive proteins (and those that can be made so) the unrivalled combination of reduced radiation damage, atomic resolution (where crystalline samples can be used) and femtosecond time resolution are ideally suited to the study of photosynthesis and the early stages of many other photochemical processes. Imaging of the water-splitting event might form one such ‘grand challenge’ project, for example. For slower processes, the mix-and-inject approach is undergoing exciting development, promising to elucidate the atomic mechanisms involved in enzymology, which may be relevant to the use of intermediate species as drug targets, as another challenge (Johnson *et al.*, 2013 ▸). The single-particle project will require much further development; however, time-resolved single-particle imaging at high resolution would at a stroke remove all of the complexities of ensemble averaging which so complicate many other methods, and perhaps reveal the large, rate-limiting conformational changes not observed by other methods. Finally, the range of new triggers under development for fast molecular imaging using an XFEL makes this a most exciting time to be involved in this rapidly growing field, with many new machines coming online worldwide in the next few years. As Humphrey Davey commented in 1806 ‘Nothing promotes the advancement of Science so much as the invention of a new instrument’.
